# New Appliances and Things Medical

**Published:** 1901-03-09

**Authors:** 


### NEW APPLIANCES AND THINGS MEDICAL.

[We shall be glad to receive, at our Office, 28 & 29 Southampton Street, Strand, London, W.C., from the manufacturers, specimens of all new preparations and appliances which may be brought out from time to time.]

### ICILMA PREPARATIONS.

(BARCLAY & SONS, LIMITED, 95 FARRINGDON STREET, LONDON, E.C.)

ICILMA water, which is claimed to be a natural product, is the basis of a considerable number of preparations which are offered for toilet use. It is apparently an oxygenised water, and hence has considerable antiseptic powers of a simple yet rational order. This water, as an application to the skin, not only removes the waste and deleterious matters which clog the pores and prevent the free action of the glands, but also at the same time acts as a tonic and mild astringent, thus enabling the integument to perform its important functions under the most favourable circumstances. For the slight blemishes and cuticular spots which are supposed to be due to the presence of noxious waste products, Icilma appears to exercise a remarkable remedial effect, probably by oxidising such substances. Icilma may be applied either as a fluid, a cream, a paste, or a soap; the ultimate effect is the same in each case, but the method of application is different, according to the indications or object desired.

### COOK'S SOAPS AND TOILET PREPARATIONS.

(EDWARD COOK & CO., LIMITED BOW, LONDON, E.)

WE have on more occasions than one drawn attention to the remarkable purity of Cook's soaps: the greatest care being exercised, not only in the manufacture, but also in the selection of the materials from which they are prepared. Being manufacturers on an enormous scale they are in a position to command the most skilled labour, as well as the most experienced chemists to direct and supervise the various processes. Among the soaps which are particularly valuable we would mention the Riviera superfatted toilet soap, an exceedingly cheap article, considering the high finish and the value of the perfume with which it is impregnated. Among the several medicated soaps, Cook's eucalyptus is to be recommended as a safe and reliable antiseptic, as well as the camphor, rosemary, and borax varieties. The hygienic tooth soap is a most serviceable and cleansing preparation for the teeth.

